# Reduction in serum sphingosine 1-phosphate concentration in malaria

**DOI:** 10.1371/journal.pone.0180631

**Published:** 2017-06-30

**Authors:** Chuchard Punsawad, Parnpen Viriyavejakul

**Affiliations:** 1 School of Medicine, Walailak University, Nakhon Si Thammarat, Thailand; 2 Tropical Diseases and Parasitic Infectious Diseases Research Group, Walailak University, Nakhon Si Thammarat, Thailand; 3 Department of Tropical Pathology, Faculty of Tropical Medicine, Mahidol University, Bangkok, Thailand; Agency for Science, Technology and Research - Singapore Immunology Network, SINGAPORE

## Abstract

Sphingosine 1-phosphate (S1P) is a lipid mediator formed by the metabolism of sphingomyelin which is involved in the endothelial permeability and inflammation. Although the plasma S1P concentration is reportedly decreased in patients with cerebral malaria, the role of S1P in malaria is still unclear. The purpose of this study was to examine the impact of malaria on circulating S1P concentration and its relationship with clinical data in malaria patients. Serum S1P levels were measured in 29 patients with *P*. *vivax*, 30 patients with uncomplicated *P*. *falciparum*, and 13 patients with complicated *P*. *falciparum* malaria on admission and on day 7, compared with healthy subjects (n = 18) as control group. The lowest level of serum S1P concentration was found in the complicated *P*. *falciparu*m malaria group, compared with *P*. *vivax*, uncomplicated *P*. *falciparum* patients and healthy controls (all *p* < 0.001). In addition, serum S1P level was positively correlated with platelet count, hemoglobin and hematocrit levels in malaria patients. In conclusions, low levels of S1P are associated with the severity of malaria, and are correlated with thrombocytopenia and anemia. These findings highlight a role of S1P in the severity of malaria and support the use of S1P and its analogue as a novel adjuvant therapy for malaria complications.

## Introduction

It was estimated that 3.2 billion people still remain at risk of malaria in 2015. According to the latest reports, 214 million cases of malaria occurred globally with estimated mortality of 438,000 deaths [1]. Malaria in humans is caused by 5 plasmodium species, namely *Plasmodium falciparum*, *P*. *vivax*, *P*. *malariae*, *P*. *ovale* and *P*. *knowlesi*. Most of the fatal cases, which predominantly occur in *P*. *falciparum* infection, are due to cerebral malaria or severe anemia, but different clinical complications such as acute kidney injury, pulmonary edema, metabolic acidosis and thrombocytopenia also exist and vary in severity and outcome, depending on the parasite species, the organ involved and the access to care [2, 3].

The pathogenicity of severe malaria infection is complex and it is regulated by both parasite and host factors. The process of cytoadhesion between infected red blood cells (RBCs) and vascular endothelium, as well as the production of inflammatory cytokines (ie. tumor necrosis factor (TNF)-α, interleukin (IL) 6, IL1β, or IL-10) are believed to contribute to the pathogenesis of the severe form of *P*. *falciparum* malaria [2, 4–6]. After malaria infection, the endothelial cells can be activated by different mechanisms such as the binding of soluble cytokines, mediated by pro-inflammatory cytokines present in host serum [7, 8], direct contact with infected RBCs, and activation induced by parasite-derived molecules such as hemozoin and glycosylphosphatidylinositol (GPI). These factors can induce endothelial injury and apoptosis which lead to increase in vascular permeability. A bioactive molecule- sphingosine 1-phosphate (S1P) has been reported to potentially regulate both endothelial permeability and inflammation. The molecule is regulated by the balance between its synthesis through sphingosine kinase (SphK) and its degradation by S1P lysase. S1P can function in autocrine or paracrine manner through plasma membrane G-protein-couples receptor (S1PR1-S1PR5) or acts directly on intracellular targets [9–11]. The potential contributions of S1PR signaling involved the modulation of vascular barrier function, vascular tone, and regulation of lymphocyte trafficking [9]. Accumulating evidences indicate that SphK/S1P signaling plays important role in the regulation of endothelium function and inflammation. Previous reports have investigated the physiological regulation of serum or plasma S1P levels in human and animal model in several diseases [12–16]

The major sources of circulating S1P are platelets [17, 18] and red blood cells (RBCs) [19]. In severe *P*. *falciparum* malaria, thrombocytopenia and anemia are common. Therefore, it is hypothesized that thrombocytopenia and anemia may lead to a reduction in S1P level in malaria. This study was designed to determine whether differences exist in the serum S1P concentrations in malaria patients and to investigate whether they are correlated with the clinical parameters, specifically parasite count, platelet count, hemoglobin (Hb) and hematocrit (Hct) levels.

## Materials and methods

### Subjects

Malaria patients admitted to the Hospital for Tropical Diseases, Faculty of Tropical Medicine, Mahidol University, Thailand, were invited to participate in this study. Patients were divided into three groups: (1) *Plasmodium vivax* malaria (n = 29), (2) uncomplicated *Plasmodium falciparum* malaria (n = 30), and (3) complicated *P*. *falciparum* malaria (n = 13). The diagnosis of malaria species was based on microscopic examination of blood smear. Blood films collected from all patients were examined by expert medical technologist and clinical diagnosis was confirmed by specialist doctor. Mixed infections *(P*. *falciparum* and *P*. *vivax*) were not detected and tested by molecular methods. Severity of *P*. *falciparum* was defined by the World Health Organization (WHO) criteria [20]. Complicated malaria was defined as patients exhibiting one or more of the following manifestations: hyperparasitemia (> 250,000 parasites/μl), hypoglycemia (glucose < 22 nmol/l), severe anemia (hematocrit (Hct) < 20% or hemoglobin (Hb) < 7.0 g/dL), or increased serum level of creatinine of more than 3.0 mg/dL. Cerebral malaria was defined as unrousable coma with positive asexual forms of *P*. *falciparum* in blood smears, with other causes of coma excluded. Eighteen healthy volunteers with no history of malaria infection were recruited as control group. Written informed consent was obtained from all patients or their close relatives before enrollment in the study, and kept in the locked file cabinet accessible only by investigators. The study protocol was approved by the Ethics Committee, Faculty of Tropical Medicine, Mahidol University (MUTM 2014-055-01, with amendment, MUTM 2014-055-02 and MUTM 2014-055-03)

### Serum sample preparation

Whole blood specimens were obtained from malaria patients on day 0 (pre-treatment- before antimalarial drug was administered) and day 7 (post-treatment) and directly collected into plastic tubes. The collected blood samples were left to stand for 15 min at room temperature (RT) to allow for clot formation, then centrifuged at 1500 g for 5 min. Subsequently, the supernatant as the serum was harvested and stored in an aliquot state at -80°C until analysis.

### Measurement of S1P levels

The serum S1P concentrations were measured by S1P competitive ELISA kit according to the manufacturer’s protocol (Echelon Biosciences, Inc., USA). Briefly, the serum was diluted (1: 10) in delipidized human sera. The diluted serum was incubated with the anti-S1P antibody in the mixing plate and incubated at RT for 60 min. After incubation, 100 μl of the reaction sample were transferred into wells of S1P coated detection plate and incubated for 60 min at RT, then washed with PBS for three times. After washing, 100 μl of streptavidin-horseradish peroxidase as the secondary detector was added in each well and incubated at RT for 30 min. The colour reaction was developed by incubation with 3,3′,5,5′ tetramethylbenzidine (TMB) substrate. Finally, the reaction was stopped with 100 μl of 1N sulfuric acid and read at OD 450 nm. S1P concentration level was expressed as μM. Each value from these indicated assays represents the mean ± SEM. All assays were carried out in triplicate.

### Statistical analysis

Statistical analysis was performed using SPSS version 17.0 software (SPSS, IL, USA) and graphs were created using Graph Pad Prism version (Graph Pad Inc, USA). Clinical variables are presented as mean and interquatile range (IQR). S1P levels are expressed as mean ± standard error of the mean (SEM). The normality of distribution was determined by the Kolmogorov-Smirnov test. Differences in levels of serum S1P between groups were compared by Mann-Whitney *U* test. Differences in levels of serum S1P within groups between day 0 and day 7 were tested by Wilcoxon signed-rank test. In addition, Spearman’s rank correlation (*r*_s_) was used to test the relationship between variables. Probability values (*p*) less than 0.05 were considered statistically significant.

## Results

### Clinical data of malaria patients

Clinical data of malaria patients and healthy controls are shown in Table 1. There was no statistically difference with regards to the age of any malaria patient groups compared with healthy subjects. Duration of illness was generally longer in complicated *P*. *falciparum* malaria patients. On admission, the mean malaria parasite count was significantly higher in the group of complicated *P*. *falciparum* malaria patients (699,780 parasites/μl) compared to uncomplicated *P*. *falciparum* (19,540 parasites/μl) and *P*. *vivax* malaria patients (9,840 parasites/μl) (all *p* < 0.001). At day 7 post-treatment, malaria parasite was absent in the peripheral blood of all malaria patients. According to the hematological parameters, hemoglobin (Hb) and hematocrit (Hct) were significantly decreased in patients with complicated *P*. *falciparuum*, uncomplicated *P*. *falciparum* and *P*. *vivax* compared to healthy controls on admission and at day 7 post-treatment (all *p* < 0.05). On admission, the mean platelet count was significantly decreased in all malaria groups compared to the healthy controls (all *p* < 0.001). However, the platelet count in all malaria groups had increased significantly to normal value at day 7 post-treatment.

**Table 1:** Clinical data of the malaria patients and healthy controls.

Characteristics	Healthy control (n = 18)	*P*. *vivax* (n = 29)		Uncomplicated *P*. *falciparum* (n = 30)		Complicated *P*. *falciparum* (n = 13)	
		Day 0	Day 7	Day 0	Day 7	Day 0	Day 7
Age (years)	27.00 (7.75)	27.00 (12.00)		24.50 (16.25)		31.00 (25.00)	
Sex (Male/Female)	9/9	29/0		26/4		12/1	
Duration of illness (days)	0	3 (2)*		5 (4)*^,^**		6 (3.5) *^,^**	
White blood cell count (/μl)	6,500 (2,275)	5,800 (1,600)*	6,400 (2,750)	5,750 (2,725)	5,500 (2,000)**	8,300 (6,350)***	8,400 (3,050)*^,^***
Hematocrit (%)	46.35 (6.33)	40.10 (6.45)*	40.20 (4.50)*	35.50 (9.05) *^,^**	31.95 (6.23) *^,^**	34.30 (13.05) *^,^**	28.70 (5.75) *^,^**
Hemoglobin (g/dL)	14.80 (2.10)	13.00 (1.65)*	12.90 (1.75)*	11.85 (3.15) *^,^**	10.35 (1.70) *^,^**	12.10 (4.50)*	9.60 (1.80) *^,^**
Parasite count (/μl)	0	9,840 (19,681)*	0	19,540 (60,055)*^,^**	0	699,780 (977,710) *^,^**^,^***	0
Platelet (x10^3^/ μl)	271 (71)	106 (55.50)*	299 (124)	76.5 (129)*	320 (174)*	26 (20.50)*^,^**^,^***	252 (196)

Results are expressed as median (IQR).

*Significant difference (*p* < 0.05) vs healthy controls,

**Significant difference (*p* < 0.05) vs *P*. *vivax* malaria,

***Significant difference (*p* < 0.05) vs uncomplicated *P*. *falciparum* malaria.

### Circulating S1P concentration in serum malaria patients

**Fig 1:**
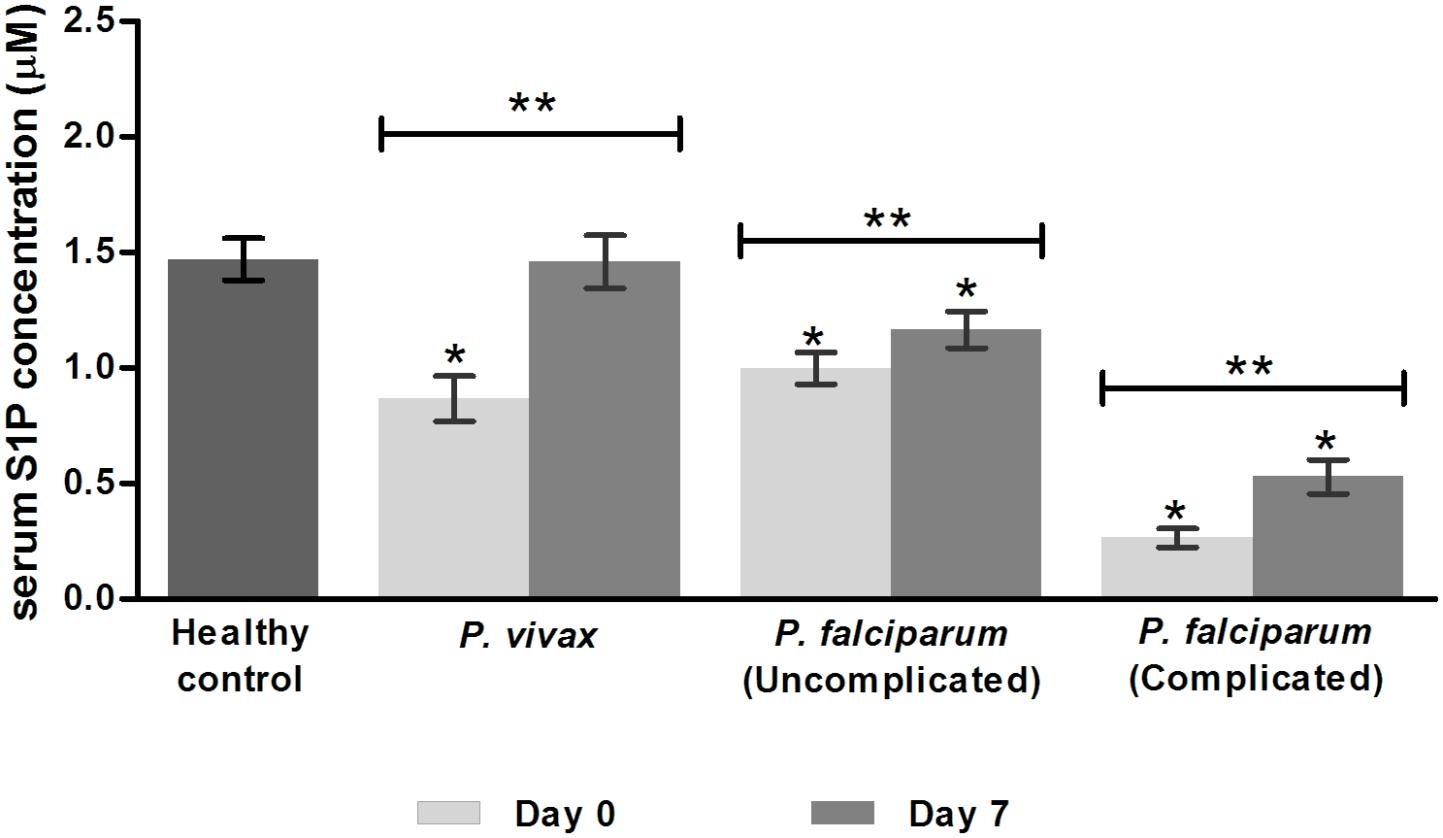
Serum S1P levels in malaria patients and healthy controls. S1P levels were measured in serum of malaria patients with *P*. *vivax* (n = 29), uncomplicated *P*. *falciparum* (n = 30) and complicated *P*. *falciparum* malaria (n = 13) on day 0 (pre-treatment) and day 7 (post-treatment) by quantitative ELISA. The healthy controls (n = 18) were used as a control group. Data are presented with mean ± SEM. *Comparison with healthy controls using Mann-Whitney *U* test. **Comparison between day 0 and day 7 using Wilcoxon signed-rank test.

Serum S1P concentrations of malaria patients and healthy controls are shown in Fig 1. On admission (day 0), serum S1P concentration was significantly reduced in all groups of malaria patients. The lowest S1P level was detected in complicated *P*. *falciparum* malaria patients (0.26 ± 0.04 μM), which showed significant differences when compared to *P*. *vivax* malaria patients (0.87± 0.10 μM), uncomplicated *P*. *falciparum* malaria patients (1.00 ± 0.07 μM), and normal controls (1.47 ± 0.09 μM) (all *p* < 0.001).

Serum S1P concentrations of all malaria groups were significantly increased on day 7 when compared with S1P levels on admission. On day 7 post-treatment, serum S1P level in *P*.*vivax* patients had increased significantly to normal level (*p* > 0.05, compared to control). However, S1P levels remained significantly decreased in patients with uncomplicated *P*. *falciparum* malaria (1.17 ± 0.04 μM) and complicated *P*. *falciparum* malaria (0.53 ± 0.07 μM), compared to the healthy controls (all *p* < 0.001).

### Correlations between serum S1P level with clinical data

**Fig 2:**
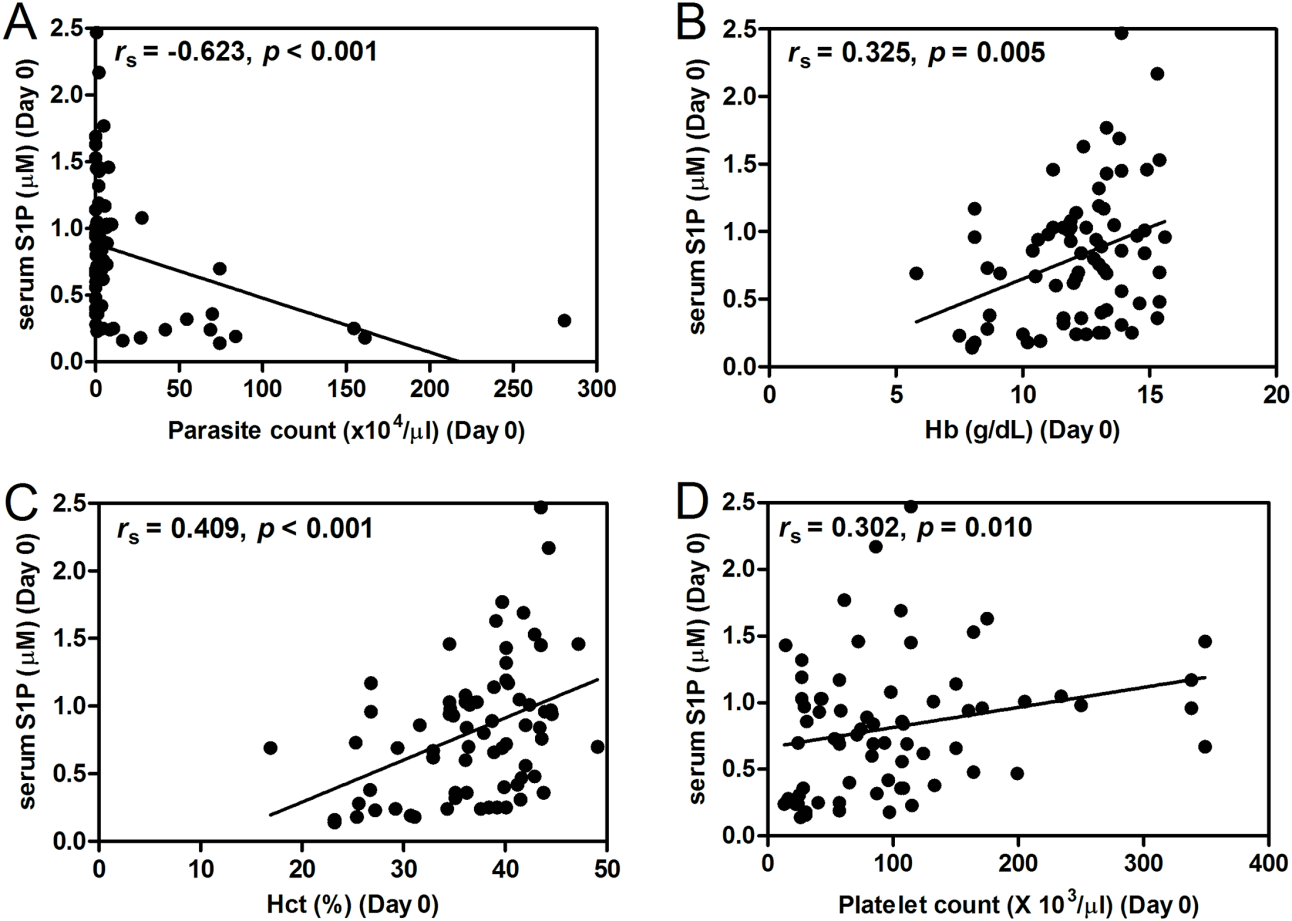
Correlations of serum S1P on admission with parasite count, hemoglobin (Hb), hematocrit (Hct), and platelet count in all malaria patients (n = 72). Data analyzed by Spearman’s rank correlation.

**Fig 3:**
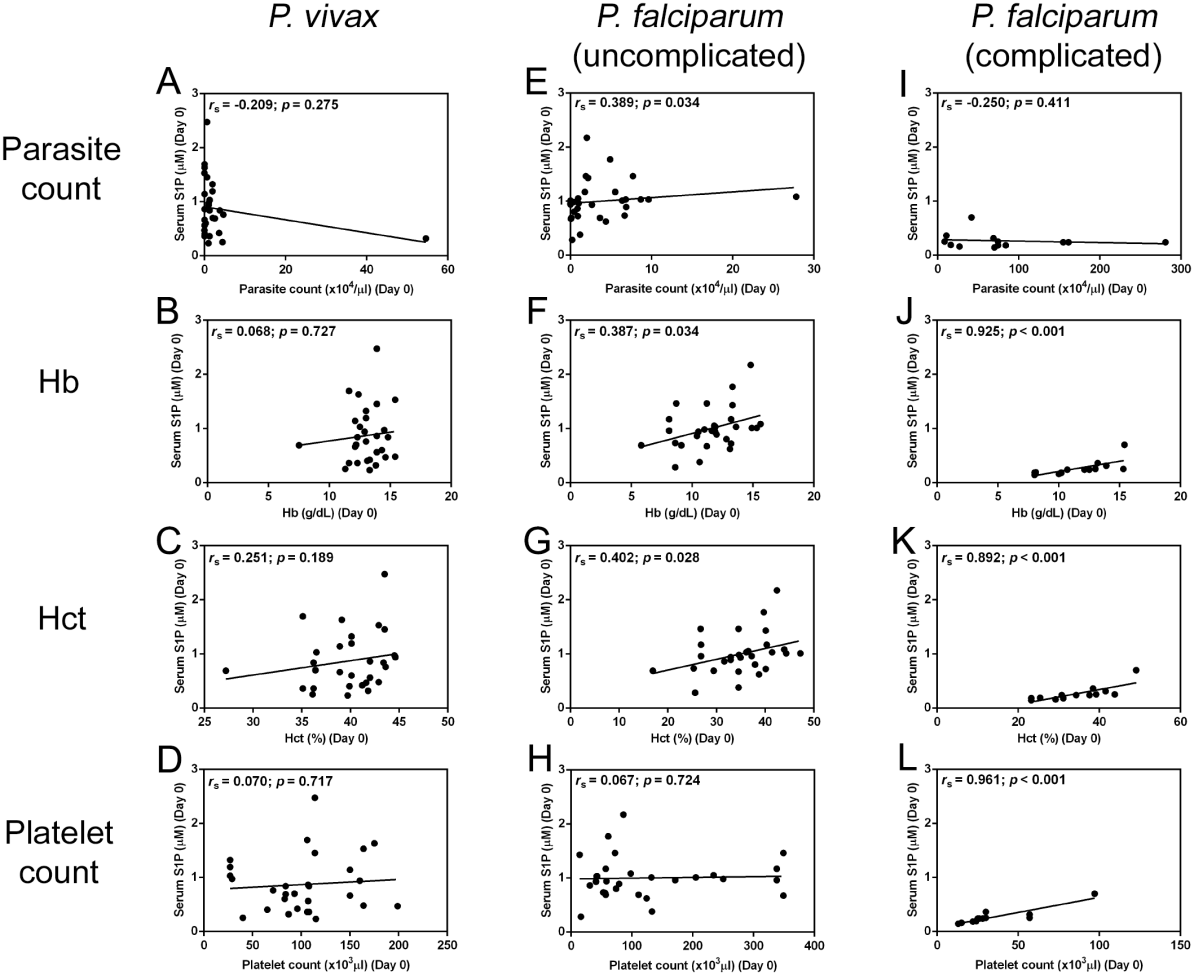
Correlation of serum S1P on admission with parasite count, hemoglobin (Hb), hematocrit (Hct) and platelet count in *P*. *vivax* (A-D), uncomplicated *P*. *falciparum* (E-H) and complicated *P*. *falciparum* (I-L) malaria patients. Data analyzed by Spearman’s rank correlation.

The number of parasite count, platelet count and the levels of Hb and Hct were important clinical parameters used to determine correlations with S1P concentration. Using pooled data, on admission, significant negative correlations was found between serum S1P concentration and parasitemia (*r*_s_ = -0.623; *p* < 0.001) (Fig 2A). Significant positive correlations was obtained between serum S1P levels and Hb (*r*_s_ = 0.325; *p* = 0.005) (Fig 2B), Hct (*r*_s_ = 0.409; *p* < 0.001) (Fig 2C) and platelet count (*r*_s_ = 0.302; *p* = 0.010) (Fig 2D). On day 7 post-treatment, serum S1P concentration showed significantly positive correlations with Hb (*r*_s_ = 0.455; *p* < 0.001) and Hct levels (*r*_s_ = 0.508; *p* < 0.001). When segregated into groups, correlations were apparent that in *P*. *vivax* group, there was no association between S1P levels and laboratory parameters (parasitemia, Hb and Hct and platelet count) at day 0 (*r*_s_ = -0.209; *p* = 0.275, *r*_s_ = 0.068; *p* = 0.727, *r*_s_ = 0.251; *p* = 0.189, *r*_s_ = 0.070; *p* = 0.717, respectively) (Fig 3A–3D) and 7 (*r*_s_ = 0.000; *p* = 0.000, *r*_s_ = -0.290; *p* = 0.127, *r*_s_ = 0.036; *p* = 0.853, *r*_s_ = 0.019; *p* = 0.920, respectively). However, in uncomplicated *P*. *falciparum*, positive correlations were obtained between S1P levels and parasitemia (*r*_s_ = 0.389; *p* = 0.034), Hb (*r*_s_ = 0.387; *p* = 0.034) and Hct (*r*_s_ = 0.402; *p* = 0.028) (Fig 3E–3H). On day 7 post-treatment, serum S1P concentration showed significantly positive correlations with Hb (*r*_s_ = 0.423; *p* = 0.020) and Hct levels (*r*_s_ = 0.429; *p* = 0.018). While in complicated *P*. *falciparum*, on the day of admission, there were strong positive correlations between serum S1P levels and Hb (*r*_s_ = 0.925; *p* < 0.001), Hct (*r*_s_ = 0.892; *p* < 0.001), and platelet count (*r*_s_ = 0.961; *p* < 0.001) (Fig 3I–3L). On day 7 post-treatment, serum S1P concentration was significantly correlated with Hb (*r*_s_ = 0.872; *p* < 0.001) and Hct levels (*r*_s_ = 0.762; *p* = 0.002). S1P level was not correlated with white blood cells in patients with severe malaria (D0: *r*_s_ = -0.011; *p* = 0.971, D7: *r*_s_ = -0.069; *p* = 0.823).

## Discussion

The main findings of our study indicated that patients with complicated *P*. *falciparum* malaria had reduced levels of serum S1P on admission and day 7 post-treatment. A decrease in S1P level had previously been documented in acute dengue infection [13], sepsis [16], chronic hepatitis C infection [21] and obesity [14]. In contrast, elevated serum level of S1P has been previously reported in autoimmune and inflammatory diseases, including juvenile-onset systemic lupus erythematosus [15] and systemic sclerosis [22]. In human malaria, S1P levels have been showed to decrease significantly in Ugandan children with cerebral malaria [12]. The current work confirms the finding in complicated *P*. *falciparum* malaria. Nevertheless, limitations in this study include (1) lack of confirmation for mixed infection of *P*. *falciparum and P*. *vivax*, (2) lack of kinetics of S1P levels in all patient groups, and (3) only pertinent clinical and laboratory findings were used to find correlation with S1P, other malaria severity parameters such as cerebral malaria, pulmonary edema, acidosis, hyperbilirubinemia were not available for analysis.

The preference for serum in S1P detection is widely discussed. During the coagulation process, platelets are activated by the clotting factors to release S1P in the serum [23]. Normally, serum S1P (0.68 μM) is higher than in plasma (0.32 μM) [24]. Previous report showed that metabolite concentrations, such as sphingomyelin were generally higher in serum than in plasma and has higher sensitivity for metabolite detection [23]. This study used serum to detect S1P since serum has an advantage of detecting low concentration of target metabolites, however, reproducibility was reported to be better in plasma than in serum [23].

Platelets and RBCs are known to play a pathological role as main sources of circulating S1P. Platelets can accumulate and release S1P after local blood coagulation [17]. Our data clearly demonstrated that serum S1P levels correlated significantly with platelet counts in malaria patients during acute infection, similar to patients with acute dengue infection [13] and sepsis [16]. It was possible that the decreased serum S1P concentration in malaria patients is caused by the reduction of platelet count and RBCs during acute infection. Our data also supported the previously documented decreased number of platelet count in malaria patients [25, 26]. It can be speculated that sphingosine kinase (SphK) in platelets may be reduced in severe *P*. *falciparum* malaria as SphK catalyzes sphingosine to S1P. Decreased in SphK activity has been reported in tuberculosis [27], bovine viral diarrhea disease and dengue virus infection [28]. Patients with complicated *P*. *falciparum* had lower platelet counts than patients infected with *P*. *vivax* malaria. Previous findings reported that S1P receptor 1 on megakaryocytes is required for the formation of proplatelet-containing cytoplasmic protrusions and releases of platelet fragments [29, 30], suggesting that S1P signaling is involved in thrombopoiesis. Although reduced in platelet count are implicated in low level of S1P in malaria patients, the involvement of S1P signaling remain incompletely characterized. However, on day 7 post-treatment, no correlation between serum S1P levels and platelet counts was observed. S1P levels fail to normalize in *P*. *falciparum* malaria (both uncomplicated and complicated malaria) after 7 days compared to *P*. *vivax* malaria suggesting a lagging time in the synthesis and released of S1P from platelets in malaria.

A reduction in Hb and Hct levels was found particularly in complicated *P*. *falciparum* which indicates the decreased in RBC volume or anemia. It is well known that both *P*. *falciparum* and *P vivax* can induce anemia during blood stages of malaria infection. Severe malarial anemia caused by *P*. *falciparum* is responsible for approximately a third of the deaths associated with the disease [31]. Anemia in malaria can be caused by a variety of pathophysiologic mechanisms. It involves direct invasion of malaria parasites into the RBCs, as well as decreased production of RBCs in the bone marrow [31, 32]. In addition to platelets, RBCs are considered to be the main source of plasma S1P. RBCs have efficiently incorporated and stored S1P and protect it from cellular degradation [19]. The suppressed bone marrow and reduction of erythroid series during malaria infection could affect S1P production. The release of S1P from RBCs may also be hampered by dysregulation of cell signaling process due to malaria infection. From the findings, serum S1P levels have positive correlations with Hb and Hct levels in *P*. *falciparum* patients on day 0 and day 7. It is possible that reduction of RBCs or anemia may contribute to low S1P levels in malaria patients. Failure of Hb and Hct to normalize after 7 days of treatment suggest a lagging time of erythropoiesis. However, the regulatory mechanism that controls blood S1P levels is not well understood. Further investigation is required to study the receptors and signaling process of S1P on RBCs in malaria patients.

Platelet level was more positively correlated with S1P concentrations in complicated *P*. *falciparum* malaria than Hb and Hct, suggesting that serum S1P might derive largely from activated platelets in prepared serum samples in malaria. The finding agrees with previous study which reported that serum S1P concentration was closely correlated with the platelet count but was very weakly correlated with the RBC count [18]. These results suggest that high concentrations of S1P may be released mainly from platelets.

S1P plays an important function is regulating pathophysiological processes involved in endothelial permeability and cytokine releases [33–35]. The reduction of S1P levels in complicated *P*. *falciparum* in this study could contribute to endothelial cell damage and affect the endothelial cell junction permeability. An experimental cerebral malaria reported that FTY720 (S1P agonist) administration improved vascular integrity in the brain, as well as reduced inflammation and reduced T-cell infiltration in the brain; and improved animal survival when used as adjuvant therapy with antimalarial treatment [12]. It was suggested that the blood communicates with blood vessels via plasma S1P to maintain vascular integrity and regulate vascular leak in mouse models [36]. Whether a reduction in the serum S1P concentration in malaria might be a simple result of the disease complications or might possibly contribute to malaria severity should be further evaluated. It is also of interest to determine whether the serum S1P concentration would be useful as a novel marker of malaria severity. To clarify this, periodic serum S1P concentrations should be measured during the acute phase of the illness and in a larger number of patients. The expression of receptors and signaling for S1P should be further investigated in RBCs, platelets and endothelial cells.

## Conclusions

Serum S1P concentration is decreased in malaria patient infected with *P*. *vivax* and *P*. *falciparum*, suggesting that S1P signaling cascade is implicated in the severity of malaria. Decreased serum S1P concentration is associated with thrombocytopenia and anemia in *P*. *falciparum* malaria as well as malaria severity. These findings can be a proof of concept to support that S1P may represent a novel drug target for adjuvant treatment for severe malaria.
